# Gα_i2_- and Gα_i3_-Deficient Mice Display Opposite Severity of Myocardial Ischemia Reperfusion Injury

**DOI:** 10.1371/journal.pone.0098325

**Published:** 2014-05-23

**Authors:** David Köhler, Vasudharani Devanathan, Claudia Bernardo de Oliveira Franz, Therese Eldh, Ana Novakovic, Judith M. Roth, Tiago Granja, Lutz Birnbaumer, Peter Rosenberger, Sandra Beer-Hammer, Bernd Nürnberg

**Affiliations:** 1 Department of Anesthesiology and Intensive Care Medicine, Institute of Experimental and Clinical Pharmacology and Toxicology, Eberhard Karls University Hospitals and Clinics, and Interfaculty Center of Pharmacogenomics and Drug Research, Tuebingen, Germany; 2 Department of Pharmacology and Experimental Therapy, Institute of Experimental and Clinical Pharmacology and Toxicology, Eberhard Karls University Hospitals and Clinics, and Interfaculty Center of Pharmacogenomics and Drug Research, Tuebingen, Germany; 3 Laboratory of Neurobiology, Division of Intramural Research, National Institute of Environmental Health Sciences, National Institutes of Health, Department of Health and Human Services, Research Triangle Park, North Carolina, United States of America; Medical School of Hannover, Germany

## Abstract

G-protein-coupled receptors (GPCRs) are the most abundant receptors in the heart and therefore are common targets for cardiovascular therapeutics. The activated GPCRs transduce their signals *via* heterotrimeric G-proteins. The four major families of G-proteins identified so far are specified through their α-subunit: Gα_i_, Gα_s_, Gα_q_ and G_12/13_. Gα_i_-proteins have been reported to protect hearts from ischemia reperfusion injury. However, determining the individual impact of Gα_i2_ or Gα_i3_ on myocardial ischemia injury has not been clarified yet. Here, we first investigated expression of Gα_i2_ and Gα_i3_ on transcriptional level by quantitative PCR and on protein level by immunoblot analysis as well as by immunofluorescence in cardiac tissues of wild-type, Gα_i2_-, and Gα_i3_-deficient mice. Gα_i2_ was expressed at higher levels than Gα_i3_ in murine hearts, and irrespective of the isoform being knocked out we observed an up regulation of the remaining Gα_i_-protein. Myocardial ischemia promptly regulated cardiac mRNA and with a slight delay protein levels of both Gα_i2_ and Gα_i3_, indicating important roles for both Gα_i_ isoforms. Furthermore, ischemia reperfusion injury in Gα_i2_- and Gα_i3_-deficient mice exhibited opposite outcomes. Whereas the absence of Gα_i2_ significantly increased the infarct size in the heart, the absence of Gα_i3_ or the concomitant upregulation of Gα_i2_ dramatically reduced cardiac infarction. In conclusion, we demonstrate for the first time that the genetic ablation of Gα_i_ proteins has protective or deleterious effects on cardiac ischemia reperfusion injury depending on the isoform being absent.

## Introduction

Cardiovascular disease (CVD) in its various forms is a major cause of morbidity and mortality worldwide. Annually more than 17 million people die from CVD which represent approximately 29% of all deaths. Among those, 7.2 million die due to heart attack resulting from coronary heart disease (WHO 2012). Once considered a disease seen predominantly in industrial nations, nowadays myocardial infarction becomes more common also in developing countries [1]. This underlines the urgent need for strategies to protect the heart from ischemic injury.

In recent years a variety of cardio-protective drugs are used in clinical practice, such as β-adrenergic blockers or adenosine which all signal *via* G-protein-coupled receptors (GPCRs) [2]. Experimentally, compounds such as adenosine, opioids and bradykinin activating Gα_i_-coupled receptors have been shown to attenuate myocardial reperfusion injury [3].

G_i_-proteins belong to the family of heterotrimeric G-proteins consisting of α, β, and γ subunits of which Gα defines the nature of the G-protein. Upon ligand binding to the GPCR, the receptor catalyzes guanine nucleotide exchange in Gα which then leads to dissociation of Gβγ from the Gα subunit. It allows both entities to interact with downstream effectors, thereby initiating intracellular signaling necessary to elicit the biological response of the cell. Aside from G_i_ three other families of heterotrimeric G-proteins are known, namely G_s_, G_q_, and G_12/13_. The G_i_-family includes three closely-related Gα members, Gα_i1-3_, each encoded by a single gene. The Gα_i1-3_-isoforms share 85–95% of amino acid sequence identity and are characterized by their sensitivity towards pertussis toxin (PTX) [4], [5]. Gα_i1_, Gα_i2_, and Gα_i3_ display overlapping expression patterns with Gα_i2_ and Gα_i3_ abundantly expressed in the cardiovascular system [6], [7]. Current research assumes that Gα_i2_ and the quantitatively minor Gα_i3_ isoform exhibit redundant physiological roles which may explain that single Gα_i2_-deficient mice show only a relatively mild, and single Gα_i3_-deficient mice no visible phenotype [8]–[10]. In line with the hypothesis that *in vivo* deletion of a single Gα_i_-isoform can functionally be at least partially compensated by remaining Gα_i_-isoforms, Gα_i2_/Gα_i3_-double-deficient mice die *in utero* at early embryonic stages [11]. However, recent studies in mice lacking Gα_i2_ or Gα_i3_ disclose distinct biological key roles of these two Gα_i_-isoforms [12]. In particular, defects of autophagic liver proteolysis, development of axial skeleton, and planar cell polarity in cochlear hair cells are solely caused by Gα_i3_-deficiency [11], [13], [14]. Contrariwise, defects in skeletal muscle growth, thrombus formation and of various immune functions of leukocytes are detectable only in Gα_i2_-deficient mice [15]–[18].

Gα_i2_ has been suggested to play a significant role in ischemia reperfusion injury of the heart while a possible involvement of Gα_i3_ has been neglected so far [19], [20]. This study was undertaken to analyze isoform-specific consequences of Gα_i_-deficiency on cardiac ischemic reperfusion injury in mice. Employing a well established and characterized murine *in vivo* model of heart ischemia and reperfusion in Gα_i_-deficient mice [21] we show that Gα_i2_-deficiency leads to massive myocardial ischemia reperfusion injury whereas Gα_i3_-deficiency is highly protective in this scenario.

## Materials and Methods

### Ethics statement

Animal experiments were conducted in strict accordance with the recommendations in the Guide for the Care and Use of Laboratory Animals (FELASA). The protocol was approved by the committee on the Ethics of Animal Experiments of local authority “Regierungspräsidium Tübingen” (permit number: TO4/10). All surgery was performed under anaesthesia as described in the respective method parts and all efforts were made to minimize suffering.

### Gα_i_-deficient mouse strains

The generation and basal phenotypic characterization of Gα_i2_-deficient and Gα_i3_-deficient mice as well as their backcrossing on a C57BL/6 background were described elsewhere [10], [11], [16], [17]. As controls wild-type C57BL/6 mice (WT) or littermates were used as indicated. Gα_i2_-deficient mice were maintained in individually ventilated cages (IVCs) and Gα_i3_-deficient mice under specific pathogen-free conditions (SPF) according to national guidelines for animal care at the animal facility of the University of Tübingen. The mice used were of either sex as we see no differences. All mice were between 8 to 12 weeks old except animals for expression analysis which were up to 14 weeks of age.

### Murine model of myocardial ischemia

Murine model of myocardial ischemic reperfusion injury was performed as described previously [21]. Briefly, after receiving anesthesia (Pentobarbital, 80 mg/kg, i.p.), mice were placed on a temperature-controlled heating table. All animals were intubated, ventilated, and left parasternal thoracotomy was performed to lay open the left coronary artery. Deep anesthesia was controlled regularly to avoid suffering of mice. Coronary artery occlusion was achieved by using the previously described hanging weight system. After reperfusion a double staining technique using triphenyl-tetrazolium chloride (TTC) to mark vital and necrotic tissue and Evans Blue staining to negatively mark the AAR was used [22]. The extent of infarct sizes were determined by calculating the percentage of infarction compared to the area at risk (AAR) from 4–5 discs per heart [21]. Each group of animal consists of at least 6 mice. Planimetric determination of infarct size and AAR was performed using the ImageJ Software version 1.44p.

### RT-PCR for transcriptional analysis

Tissue or whole blood cells were homogenized; RNA was isolated, and transcribed into cDNA. Transcriptional expression levels were measured using real-time reverse transcription polymerase chain reaction (iCycler CFX 96; Bio-Rad Laboratories, Munich, Germany) and normalized to two house-keeping genes, namely β-actin and GAPDH. To detect β-actin, GAPDH, Gα_i2_ and Gα_i3_ mRNA levels, following primers were used: GAPDH sense 5′-cga gaa tgg gaa gct tgt cat c-3′; GAPDH antisense 5′-cgg cct cac ccc att tg-3′; β-actin sense 5′-ctc tcc ctc acg cca tcc tg-3′; β-actin antisense 5′-tca cgc acg att tcc ctc tca g-3′; Gα_i2_ sense 5′-gcc aac aag tac gac ggc a-3′; Gα_i2_ antisense 5′-gta tct ctc acg ctt ctt gtg ct-3′; Gα_i3_ sense 5′-atg aac cga atg cat gaa agc a-3′; Gα_i3_ antisense 5′-ttt ggt gtc agt ggc aca ggt a-3′.

### Immunoblot detection of Gα_i_ proteins

After homogenization of tissue, samples were resuspended in RIPA buffer, and protein concentrations were measured by standard BCA method following the manufacturers' instructions (Thermoscientific, Illinois, USA). Protein amounts are indicated in the figure legends. Proteins were loaded on either urea-supplemented or 10% SDS polyacrylamide gels and blotted onto nitrocellulose membranes as described. The antibodies detecting Gα_i_ proteins, i.e. anti-Gα_com_, anti-Gα_i2_, and anti-Gα_i3_ were previously described [17], [23]. Loading conditions were controlled by GAPDH. A horseradish peroxidase (HRP)-conjugated anti-rabbit IgG antibody served for immunodetection (Santa Cruz Biotechnology, Inc, USA, Santa Cruz). Immunoreactive bands were visualized by using an ECL detection system (GE Healthcare, Braunschweig, Germany). The levels of Gα_i2_ and Gα_i3_ in different organs from WTs; in heart tissue of WT, Gα_i2_ *^-/-^*, and Gα_i3_ *^-/-^* mice; and in heart tissue at different time points at and after ischemic events were quantified by densitometric analysis using Image J 1.44p after normalizing to GAPDH level.

### Immunofluorescence staining of myocardial tissue

Untreated hearts of WT, Gα_i2_ *^-/-^*, and Gα_i3_ *^-/-^* mice and ischemic hearts (60 min) of mice after different reperfusion condition treatments (0, 60 and 120 min reperfusion) were excised and immediately frozen in Tissue-Tek® (Sakura Finetek, Netherland, Leiden). 0.5 µm thick cryostat sections were mounted on slides, fixed in 4% formaldehyde for 30 min, permeabilized with 0.1% Triton® X-100 (AppliChem, Germany, Darmstadt) for 10 min and blocked with 5% BSA in PBS for 45 min. For immunodetection of G-proteins in heart tissue, the previously described Gα_i_ antibodies, i.e. anti-Gα_i2_ and anti-Gα_i3_, were used. DAPI (Invitrogen, USA, Oregon) was applied for nuclei detection. Gα_i2_ and Gα_i3_ signals were visualized with an Alexa 488-conjugated mouse anti-rabbit IgG (Invitrogen, USA, Oregon). The fluorescence imaging was performed with an Axiophot Zeiss microscope (Zeiss, Jena) using a digital camera with AxioVision 4.8 software.

### Data analysis

Statistics were performed using one-way ANOVA with Bonferroni post test to determine group differences or unpaired student t test where appropriate. A value of P<0.05 was considered to be statistically significant.

## Results

In order to get insights into the individual role of the two major Gα_i_-proteins of the cardiovascular system, i.e. Gα_i2_ and Gα_i3_, in the development of myocardial ischemia reperfusion injury (MIRI) we studied Gα_i2_ *^-/-^* and Gα_i3_ *^-/-^* mice in comparison to wild type controls in an acute murine model of heart ischemia and reperfusion [21].

### Expression of Gα_i2_ and Gα_i3_ in murine organs

**Figure 1:**
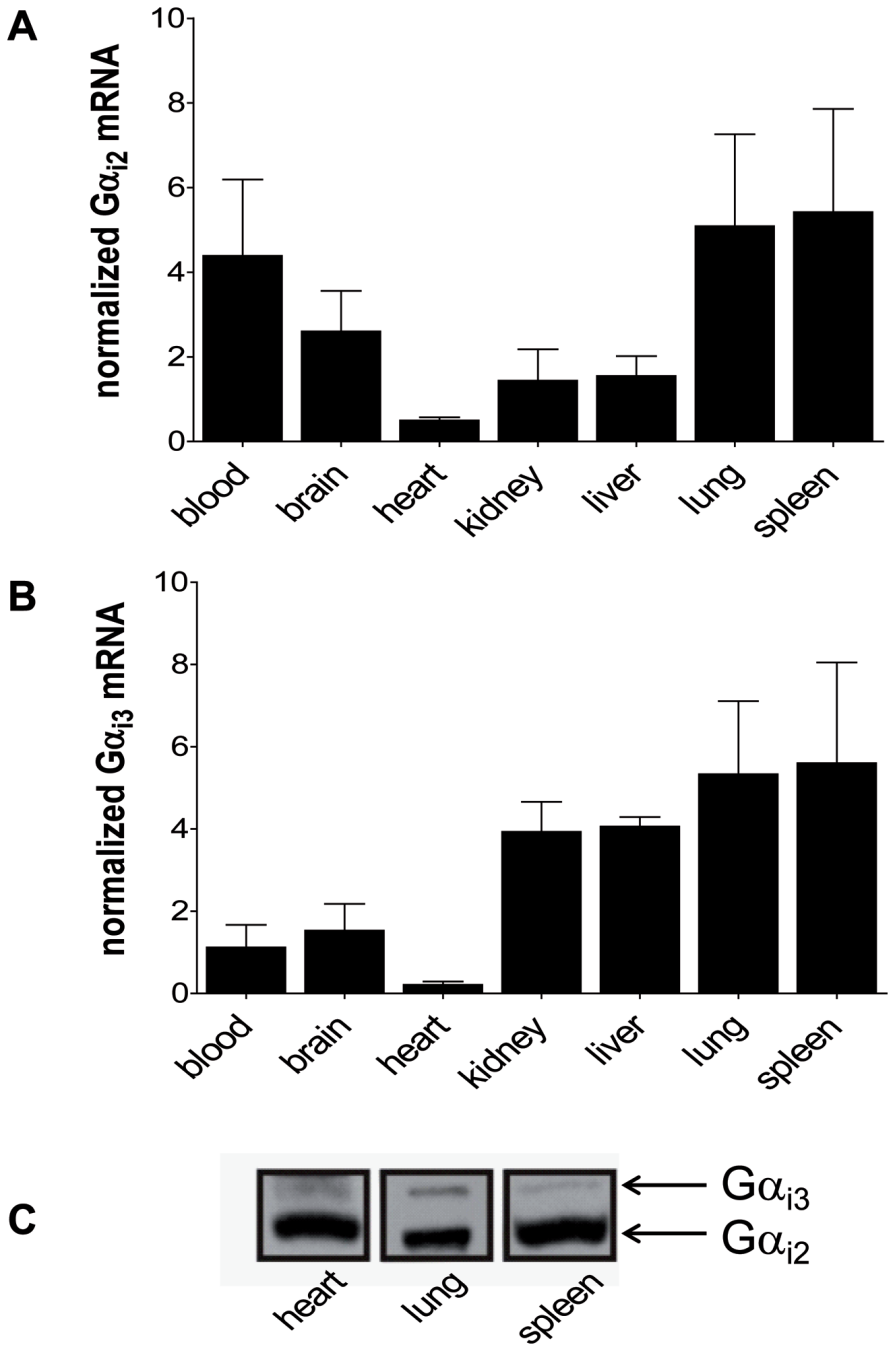
Expression of the two Gα_i_-isoforms. RT-PCR analysis of various mouse organs. Transcriptional levels of Gα_i2_ (**a**) and Gα_i3_ (**b**) were normalized to β-actin and GAPDH. **c**. Immunoblot analysis of heart lysates from wildtype male mice with a Gα_common_ antibody following urea supplemented SDS-PAGE and western blotting.

First, we compared expression levels of murine Gα_i2_ and Gα_i3_ in the heart and various other organs (Figure 1a). In the heart, both Gα_i_-isoforms were detected on transcriptional and protein level (Figure 1a–c). Although low transcript levels were evident as compared to all other organs tested, significant protein expression was detectable in immunoblot analysis. Notably, as described for many organs and tissues [6], [11], [17] Gα_i2_ is also the predominant isoform in the heart, although we found significant levels of Gα_i3_ (Figure 1c).

### Up regulation of Gα_i_-proteins during myocardial IR

**Figure 2:**
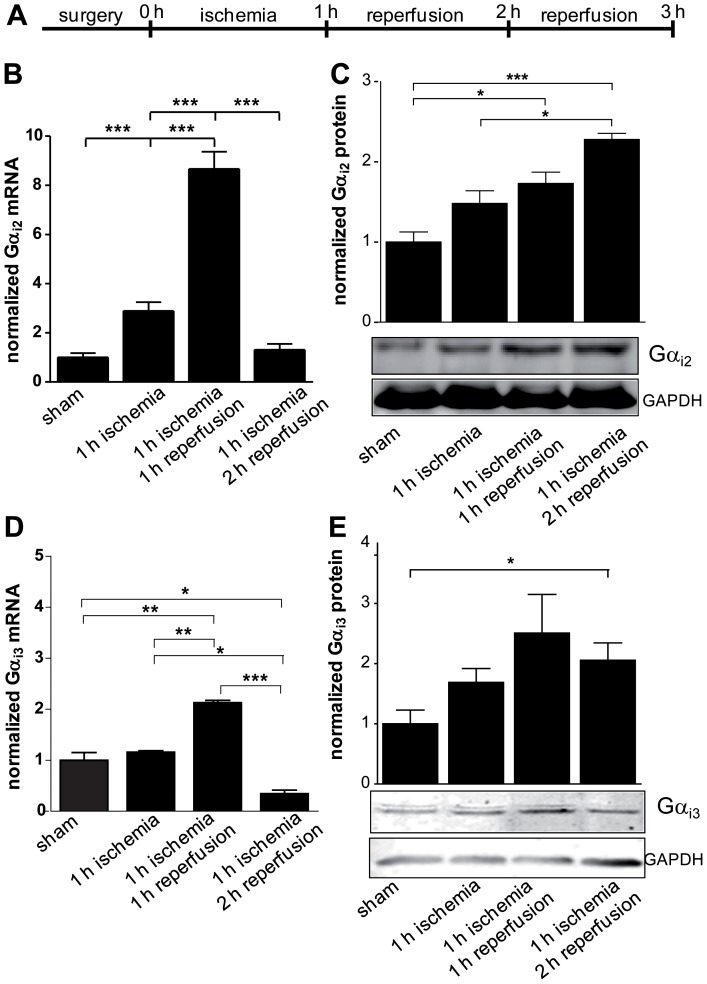
Increased expression of Gα_i2_ and Gα_i3_ during IR-injury. **a**. Schedule of the IR model with one hour ischemia followed by one or two hour reperfusion. **b**. Surgeries were performed as indicated with C57BL/6 male mice (WT). After the procedures AAR was excised and transcript levels of Gα_i2_ were determined by quantitative PCR. **c**. Protein expression of Gα_i2_ was analyzed by immunoblotting using a Gα_i2_-specific antibody. Protein amounts were normalized to GAPDH. Protein amounts loaded were 60 µg. Shown are representative images. **d**. Transcript levels of Gα_i3_ after surgeries as described in (a) were determined by quantitative PCR. **e**. Protein expression of Gα_i3_ was analyzed by immunoblotting using Gα_i3_-specific antibodies. Protein amounts were normalized to GAPDH. Protein amounts loaded 60 µg. Shown are representative images. Data are shown as mean ± SEM (n = 3); statistic was calculated with one-way ANOVA, with Bonferroni post test; *P≤0.05; **P≤0.01; ***P≤0.001 as indicated.

In accordance with its predominant expression, Gα_i2_ has been reported to play an important role during ischemic injury [19]. Moreover, a recent study showed that enhancement of Gα_i2_ signaling through loss of its negative regulation by RGS proteins protects the heart from ischemic injury [20]. Therefore, we wondered whether the different phases of myocardial ischemia reperfusion regulate expression levels of Gα_i2_ since transcript levels were obtained 72 hrs after ischemia [19]. To address this question, the mice were exposed to a one hour period of ischemia followed by two hours of reperfusion (Figure 2a). At defined time points mice were sacrificed and their hearts analyzed. The cardiac tissue from the area at risk (AAR) was excised and transcript and protein expression levels of Gα_i2_ (Figure 2b and c) and Gα_i3_ (Figure 2d and e) were measured and compared to samples from sham-operated controls. The Gα_i2_-specific mRNA was up regulated more than threefold after one hour of ischemia while there was no significant change in the protein level. In the following early phase of reperfusion Gα_i2_ transcripts peaked with an eightfold increase with subsequent more than twofold increase in the protein level during late phase of reperfusion (Figure 2b and c). Similarly, Gα_i3_ mRNA was also regulated during ischemia and reperfusion time in a similar manner but less intense (Figure 2d). Interestingly, protein levels increased statistically significant more than twofold during reperfusion phase (Figure 2e). To exclude that this enhanced expression is due to a massive influx of PMNs which highly express Gα_i2_ and Gα_i3_ the AAR sections were stained with anti-Gα_i_- and subsequently with anti-CD15-antibodies (Figure S1). In fact, while leukocytes infiltrated into the heart tissue during the reperfusion phase a clearly enhanced Gα_i2_- and Gα_i3_-specific staining of cardiac tissue was evident. Taken together, the results may indicate that cardiac Gα_i2_ and Gα_i3_ play similar roles during ischemia reperfusion injury in the heart.

### Gα_i2_-deficiency aggravates whereas Gα_i3_-deficiency ameliorates IR-injury

**Figure 3:**
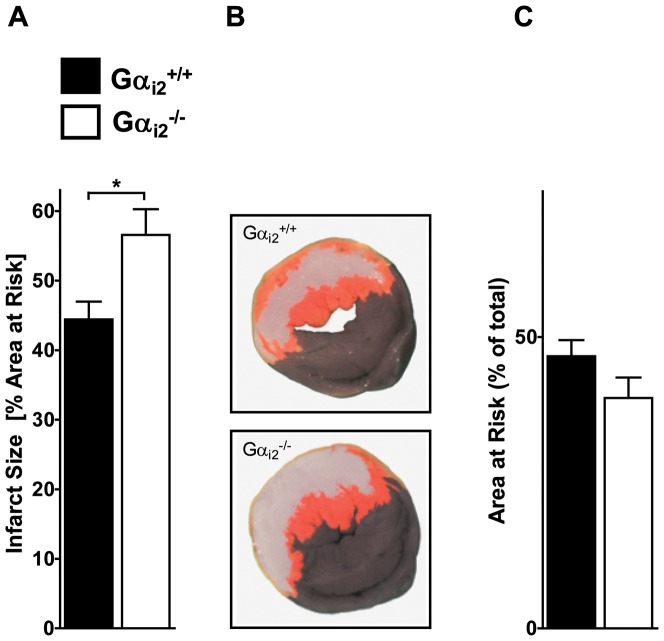
Gα_i2_-deficiency aggravates IR-injury. **a**. Gα_i2_^+/+^ (n = 6) and Gα_i2_^-/-^ (n = 7) mice were exposed to one hour ischemia followed by two hours reperfusion. Hearts were stained with Evans Blue to determine the AAR and TTC to mark vital tissue (red) and necrotic tissue (white). Subsequently, infarct size was calculated as percentage of AAR (for details see Method's section). **b**. Representative heart slice of Gα_i2_^+/+^ and Gα_i2_^-/-^ mice are shown. These heart discs have an AAR of 50% (Gα_i2_^+/+^) and 50% (Gα_i2_^-/-^). The infarcted area was 41% (Gα_i2_^+/+^) and 58% (Gα_i2_^-/-^). **c**. Quantification of AAR as a percentage of the total heart disc (p = 0.14). Data in (a) and (c) are shown as mean ± SEM; statistic was calculated with t-test; *P≤0.05 as indicated.

**Figure 4:**
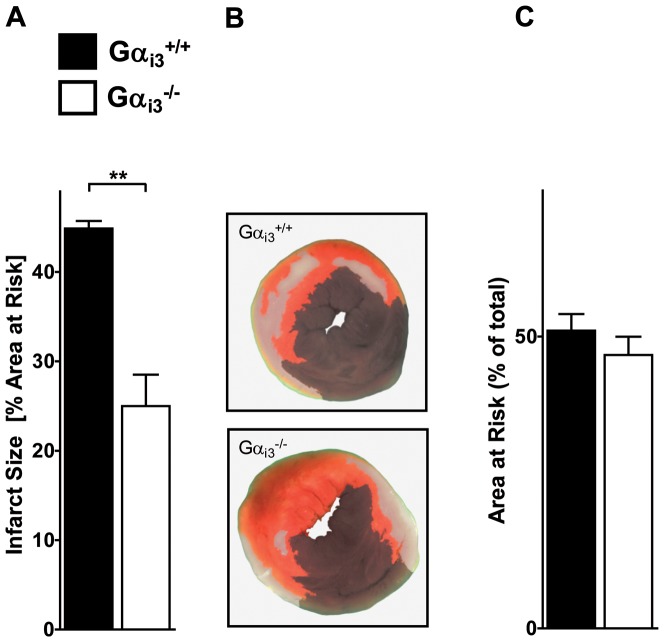
Gα_i3_-deficiency ameliorates IR-injury. **a**. Gα_i3_^+/+^ (n = 7) and Gα_i3_^-/-^ (n = 6) mice were exposed to one hour ischemia followed by two hours reperfusion. Hearts were stained with Evans Blue to determine the AAR and TTC to mark vital tissue (red) and necrotic tissue (white). Subsequently, infarct size was calculated as percentage of AAR (for details see Method's section). **b**. Representative heart slice of Gα_i3_^+/+^ and Gα_i3_^-/-^ mice are shown. These heart discs have an AAR of 42% (Gα_i3_^+/+^) and 57% (Gα_i3_^-/-^). The infarcted area was 43% (Gα_i3_^+/+^) and 28% (Gα_i3_^-/-^). **c**. Quantification of AAR as a percentage of the total heart disc (p = 0.35). Data in (a) and (c) are shown as mean ± SEM; statistic was calculated with t-test; **P≤0.01 as indicated.

To challenge the concept of redundancy of the two Gα_i_ isoforms in IR-injury, we performed regional myocardial ischemia reperfusion in Gα_i2_- (Figure 3) and Gα_i3_-deficient mice (Figure 4). In this model the infarct size is determined by comparing the area of infarction within the area at risk (AAR) [21]. Vital and necrotic tissues within the AAR were identified by double staining with TTC and Evans Blue, respectively. The degree of myocardial destruction was calculated as percentage of infarcted myocardium to AAR. In this experimental setting WT mice show expected infarct sizes of 44.4±2.6%, whereas Gα_i2_ *^-/-^* mice displayed significantly increased infarct areas of 56.6±3.7% (Figure 3a). To illustrate the infarct size in a more visual fashion, one representative heart disc of each WT and Gα_i2_ *^-/-^* mice is depicted (Figure 3b). Concurrently, the area at risk was not significantly different between groups (Figure 3c). This supports and extends the previously described protective role of Gα_i2_ in ischemia reperfusion [19], [20].

Surprisingly, in Gα_i3_ *^-/-^* mice the extent of damage after myocardial IR-injury was dramatically decreased. Gα_i3_ *^-/-^* mice exhibited strong reduction in infarcted areas (25.0±3.5%) compared to controls (44.9±0.8%; Figure 4a) while there was no significant change in AAR (Figure 4c). Again, one representative heart disc of both groups is pictured (Figure 4b). These data reveal an up to now unknown protective mechanism in mice against IR injury in the absence of Gα_i3_. Therefore, in contrast to current thinking, our data suggest that Gα_i2_ and Gα_i3_ play opposite instead of redundant roles in IR injury.

### Increased expression of Gα_i2_ in Gα_i3_-deficient mice and vice versa

**Figure 5:**
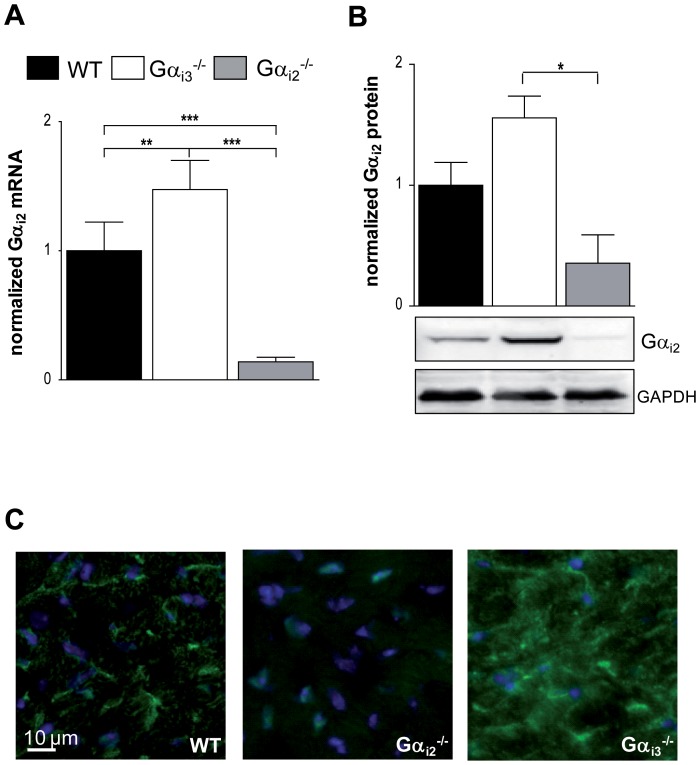
Expression of Gα_i2_ in heart tissue of Gα_i3_-deficient mice. **a**. mRNA levels of Gα_i2_ in hearts of wildtype (WT), Gα_i2_-deficient (Gα_i2_^-/-^) and Gα_i3_-deficient male mice (Gα_i3_^-/-^) (n = 4). **b**. Representative immunoblots of heart from WT, Gα_i2_^-/-^ and Gα_i3_^-/-^ male mice detected with a Gα_i2_-specific antibody. GAPDH was used to normalize the amount of protein. Protein amounts loaded 60 µg. The graph depicts the densitometric analysis (n = 3). **c**. Immunohistochemical staining of Gα_i2_ in heart tissue of WT, Gα_i2_^-/-^ and Gα_i3_^-/-^ mice. Representative pictures are shown. Scale bar: 10 µm. Data are shown as mean ± SEM; statistic was calculated with one-way ANOVA, with Bonferroni post test; *P≤0.05; **P≤0.01; ***P≤0.001 as indicated.

**Figure 6:**
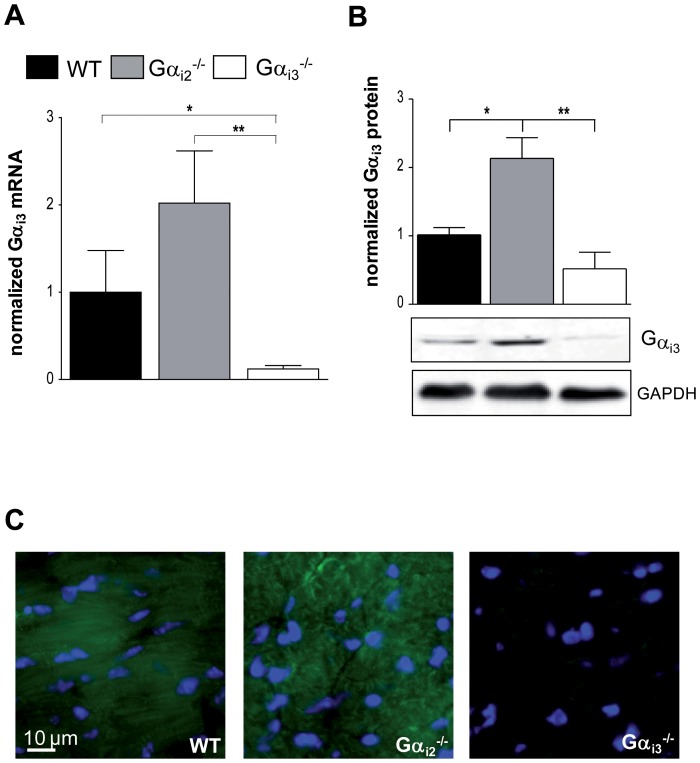
Expression of Gα_i3_ in heart tissue of Gα_i2_-deficient mice. **a**. mRNA levels of Gα_i3_ in hearts of wildtype (WT), Gα_i2_-deficient (Gα_i2_^-/-^) and Gα_i3_-deficient male mice (Gα_i3_^-/-^) (n = 3). **b**. Representative immunoblots of heart from WT, Gα_i2_^-/-^ and Gα_i3_^-/-^ male mice detected with a Gα_i3_-specific antibody. GAPDH was used to normalize the amount of protein. Protein amounts loaded 60 µg. The graph depicts the densitometric analysis (n = 3). **c**. Immunohistochemical staining of Gα_i3_ in heart tissue of WT, Gα_i2_^-/-^ and Gα_i3_^-/-^ mice. Representative pictures are shown. Scale bar: 10 µm. Data are shown as mean ± SEM; statistic was calculated with one-way ANOVA, with Bonferroni post test; *P≤0.05; **P≤0.01; ***P≤0.001 as indicated.

Previously, we detected an up regulation of the remaining isoform in different murine Gα_i_-deficient tissues and cells for either Gα_i2_ or Gα_i3_ [11], [17]. This is thought to represent an important mechanism contributing to functional redundancy and prompted us to ask whether the hearts of the knockout-mice lack compensatory up regulation of the remaining Gα_i_-isoform. In a previous attempt we have analyzed hearts from knock out-mice using high resolution SDS-PAGE in combination with Gα_common_ antibodies [6]. However; only partial resolution of Gα_i3_ from Gα_i2_ limited the value of the densitometric analysis. In particular, the upregulation of Gα_i3_ in the absence of Gα_i2_ might have been overestimated. To approach this question in a more rigorous way, we measured mRNA and protein levels of Gα_i2_ in the cardiac tissue from Gα_i3_ *^-/-^* mice and *vice versa* (Figure 5 and 6). For assessment normalized transcript levels and immunoblot intensity of target proteins were compared to the housekeeping protein GAPDH. Isoform-specific antibodies were used for individual detection of Gα_i2_ or Gα_i3_ [11], [17]. Both approaches showed significantly higher expression levels of Gα_i2_ in Gα_i3_ *^-/-^* mice (Figure 5a and b) and *vice versa* Gα_i3_ in Gα_i2_ *^-/-^* mice (Figure 6a and b). In Gα_i2_ *^-/-^* mice which lack the predominant Gα_i_ isoform, Gα_i3_ was up regulated more than twofold in cardiac tissue, and in Gα_i3_ *^-/-^* mice Gα_i2_ expression levels were increased by 50%. To strengthen our finding on up regulated protein expression in either knockout mouse model, we performed immunofluorescence staining of murine heart tissue sections (Figure 5c and 6c). Validation of the primary and secondary antibodies is shown in Figure S2. In accordance with the RT-PCR and Western blot results in either case signals of the remaining Gα_i_-isoform were highly increased in heart tissue of the knock-out counterpart.

Therefore, we conclude that the compensatory up regulation of the remaining Gα_i_ isoform did not mask the phenotype seen in the knock-out models.

## Discussion

The role of G_i_-protein-dependent receptor signaling in the cardiovascular system is still a matter of intense investigations. A variety of therapeutics acting on G_i_-PCRs is currently in use for regulation of heart function and protection. Therefore, cardiovascular G_i_-PCRs are the most targeted receptors in the pharmacological treatment of cardiac diseases [2], [24]. In principle all these receptors can couple to two Gα_i_-isoforms, i.e. Gα_i2_ and Gα_i3_. However, current thinking implies only Gα_i2_ as the isoform responsible for eliciting biological effects whereas a role for Gα_i3_ is neglected in this scenario. Hence, the aim of our study was to focus on a role for Gα_i3_ in cardiac ischemia.

Here, we show for the first time that the absence of Gα_i2_ or Gα_i3_ have opposite effects on the severity of myocardial IR injury in knockout mice. In particular, Gα_i2_-deficiency led to enhanced myocardial infarct size whereas the absence of Gα_i3_ was highly protective. Whereas the first observation confirms and extends previous studies [19], [20], the latter finding was unexpected. The increased infarct size visible in Gα_i2_-deficient mice underlines a protective role of Gα_i2_ signaling which was reported in previous studies making use of different experimental approaches. For instance, *in vivo* administration of PTX being considered a functional pan-G_i_-inhibitor in combination with an infarct model demonstrated a cardio-protective effect of these G-proteins in rat hearts [25]. We performed similar experiments using our acute mouse model of 60 min. of regional myocardial ischemia followed by 120 min. reperfusion *in vivo* (Figure S3 and Methods S1). Interestingly, infarct sizes of the PTX-treated animals were even more pronounced as compared to those seen in Gα_i2_-deficient mice, i.e. 67.0±4.8% *vs.* 56.6±3.7%, respectively, whereas the values for the controls in either group were almost the same (42.3±2.2% *vs.* 44.4±2.6%). The latter data argue for a reliable procedure as indicated by similar values in both control groups. PTX modifies Gα_i_-proteins by ADP-ribosylation of a cystein residue in the extreme C-terminus of sensitive Gα_i_-proteins. In the afore-mentioned study in rats [25] the degree of PTX-induced *in vivo* ADP-ribosylation of cardiac Gα_i_-proteins was assessed by employing a radioactive *in vitro* approach. Interestingly, this analysis revealed that only a small subpopulation of G_i_-proteins in the myocardial membrane was PTX-modified. This is a phenomenon we also see in our studies (data not shown). Since PTX modifies Gα_i_-proteins with different efficiency, it cannot be excluded that PTX acted in a rather isoform selective way [26]. Moreover, different cells and tissues may exhibit variable sensitivity and kinetics towards PTX. Therefore it remains unclear which Gα_i_-isoforms in which tissues and organs have contributed to the observed cardio-protective effect. Another study also targeted the interaction of GPCRs with cardiac G_i_-proteins in a more specific approach [19]. Mice were created with a transgene expressing an inhibitory carboxyl-terminal 63 amino acid peptide of Gα_i2_ in cardiac tissue acting in a dominant negative fashion. These mice, when subjected to ischemia/reperfusion induced heart injury, demonstrated an exacerbated ischemic injury as compared to controls. Although the effects of the inhibitory Gα_i2_-minigene on G_i_-dependent signaling pathways were significant, the contribution of the Gα_i2_- and Gα_i3_-specific pathways to the observed cardio-protective effect was not investigated. In a recent paper a complementary genetic approach to study the effect of Gα_i2_-signaling on cardiac ischemia *in vitro* was described [20]. Knock-in mice were examined in which the endogenous Gα_i2_ gene was replaced with an RGS-insensitive G184S Gα_i2_ mutant that was unable to interact with RGS proteins. This resulted in an enhancement of Gα_i2_ signaling by reversal of its negative regulation by RGS proteins thereby protecting the heart from ischemic injury. Although this study was in accordance with the concept of Gα_i2_-dependent protection of the heart, it ignored a possible role of Gα_i3_. Moreover, these mice showed a dramatic and complex phenotype affecting the heart and several other organs which may produce secondary effects on heart function and resulting in premature death [27].

Similar concerns have been raised about the Gα_i2_ knockout model that we have used in our current study. Initially, these mice have been reported to display a histopathological phenotype resembling ulcerative colitis and adenocarcinoma of the colon [10]. However, when these mice were housed under pathogen-free conditions no obvious signs of intestinal inflammation were visible during the course of the study and they did not show the previously reported lethality phenotype [17]. This allowed us to specifically study the roles of the two Gα_i_-isoforms in cardiac ischemia injury *in vivo*.

Surprisingly, mice lacking Gα_i3_ showed a significantly reduced infarct size following IR injury. It was intriguing that the deletion of one Gα_i_ isoform results in the up regulation of the remaining ones. In fact, we detected an up regulation in heart tissue; a phenomenon we have observed previously in all tissues and cells we analyzed so far [6], [11], [17]. As a consequence, the particular knock-out model exhibits two important features, i.e. the deletion of the target Gα_i_-isoform and the enhanced expression of the remaining ones. Therefore the deleterious or protective effect might not only be the result of the loss of one isoform but also the enhanced signaling of the remaining ones. For example, the increased infarct size seen in Gα_i2_-deficient mice could either be due to missing Gα_i2_ or over-expressed Gα_i3_. Conversely, the reduced infarct size in Gα_i3_-deficient mice could either be due to the over-expressed Gα_i2_ or absent Gα_i3_. In that respect, it will be interesting to re-evaluate the previous studies discussed above [19], [20]. The main conclusions from these studies were to attach a predominant role of Gα_i2_ in ischemia reperfusion injury. However, these studies ignored that an altered Gα_i2_ signaling could affect Gα_i3_ expression – as observed here and in previous studies – and signaling. This is of special importance since Gα_i3_ has been shown to play crucial roles in both, its GDP-bound and GTP-bound form [28], [29]. The current view is that Gα_i2_ and Gα_i3_ have largely overlapping roles. Some of our recent data contradict such a claim, showing that the absence of the minor Gα_i3_ isoform cannot be compensated by the remaining Gα_i2_ isoform [11], [13], [14]. G-protein signaling pathways come in at least two different shapes: a canonical and a non-canonical pathway which may mechanistically establish non-redundant distinct functions [29]. Future works have to concentrate on solving this question.

The current study displays intriguing and highly significant differences between the two Gα_i_-isoforms albeit it employed a relatively small number of animals. One obvious limitation is the fact that global knockout animals, which lack the respective Gα_i_-isoform in every tissue or organ, were studied. For future directions of research, in particular additional tools are required to decipher the specific functions of the two Gα_i_ isoforms in cardiac and non-cardiac cells, e.g. cardiomyocytes, endothelial or immune cells. Ideally, experimental approaches may include detailed analyses of tissue-specific mouse models where the Gα_i_ gene of interest is deleted in a constitutive or inducible manner. This allows elucidating the individual contribution of the Gα_i_-isoforms to the ischemic reperfusion injury in the heart. Furthermore with this approach an up regulation of the remaining isoform may be prevented. Whereas an appropriate Gα_i2_-model is available [13] the corresponding Gα_i3_-mouse model has not been created so far.

In conclusion, we provide strong evidence that both the deficiency for Gα_i2_ and for Gα_i3_ has profound and opposite effects on IR injury in mice. This may open the rationale to develop biased G_i_PCR drugs which may allow a different regulation of Gα_i2_ and Gα_i3_ by the same receptor.

## Supporting Information

Figure S1 PMN infiltration in heart tissue during IR-injury. Surgeries in WT mice were performed as indicated. To stain infiltrated neutrophils, a source for the level changes in Gα_i_ protein expression, immunohistochemistry with an anti-CD15 antibody was performed. Additionally, tissue was stained with **a**. Gα_i2_- and **b**. Gα_i3_-specific antibodies. Representative images are shown. Scale bar  = 10 µm.
(TIF)

Figure S2 Control staining to test antibody specificity. To rule out unspecific binding of the used antibodies in heart tissue control staining were performed as follow. **a**. Staining of WT tissue with IgG antibody. **b**. Heart tissue from Gα_i2_^-/-^ mice was stained with anti-Gα_i2_ antibody. **c**. Heart tissue from Gα_i3_^-/-^ mice was stained with anti-Gα_i3_ antibody. Representative images are shown. Scale bar  = 10 µm.
(TIF)

Figure S3 PTX treatment aggravates IR injury. **a**. WT mice were either injected i.p. with vehicle (n = 6) or Pertussis toxin (PTX)(see Methods S1) (n = 6) and 48 hours later exposed to one hour ischemia and one hour reperfusion. Hearts were counterstained with Evans Blue to determine the AAR and TTC to mark vital tissue (red) and necrotic tissue (white). Subsequently, infarct size was calculated as percentage of AAR. **b**. Representative heart slice of WT mice treated with NaCl or PTX are shown. These heart discs have an infarcted area of 46% (WT+NaCl) and 69% (WT+PTX). Data in (a) are shown as mean ± SEM; statistic was calculated with t-test; ***P≤0.001 as indicated.
(TIF)

Methods S1 Pertussis Toxin treatment.
(DOCX)
