# Correction: Gα_i2_- and Gα_i3_-Deficient Mice Display Opposite Severity of Myocardial Ischemia Reperfusion Injury

**DOI:** 10.1371/journal.pone.0119092

**Published:** 2015-03-05

**Authors:** The PLOS ONE Staff

There is an error in affiliation 1 for authors David Köhler, Claudia Bernardo de Oliveira Franz, Judith M. Roth, Tiago Granja, Peter Rosenberger. Affiliation 1 should be: Department of Anesthesiology and Intensive Care Medicine, Eberhard Karls University Hospitals and Clinics, Tuebingen, Germany.
