# Supplementary figures and images for: Gαi2- and Gαi3-Deficient Mice Display Opposite Severity of Myocardial Ischemia Reperfusion Injury
Source: PLoS One. 2014 May 23;9(5):e98325. doi: 10.1371/journal.pone.0098325 (PMC4032280; doi:10.1371/journal.pone.0098325)

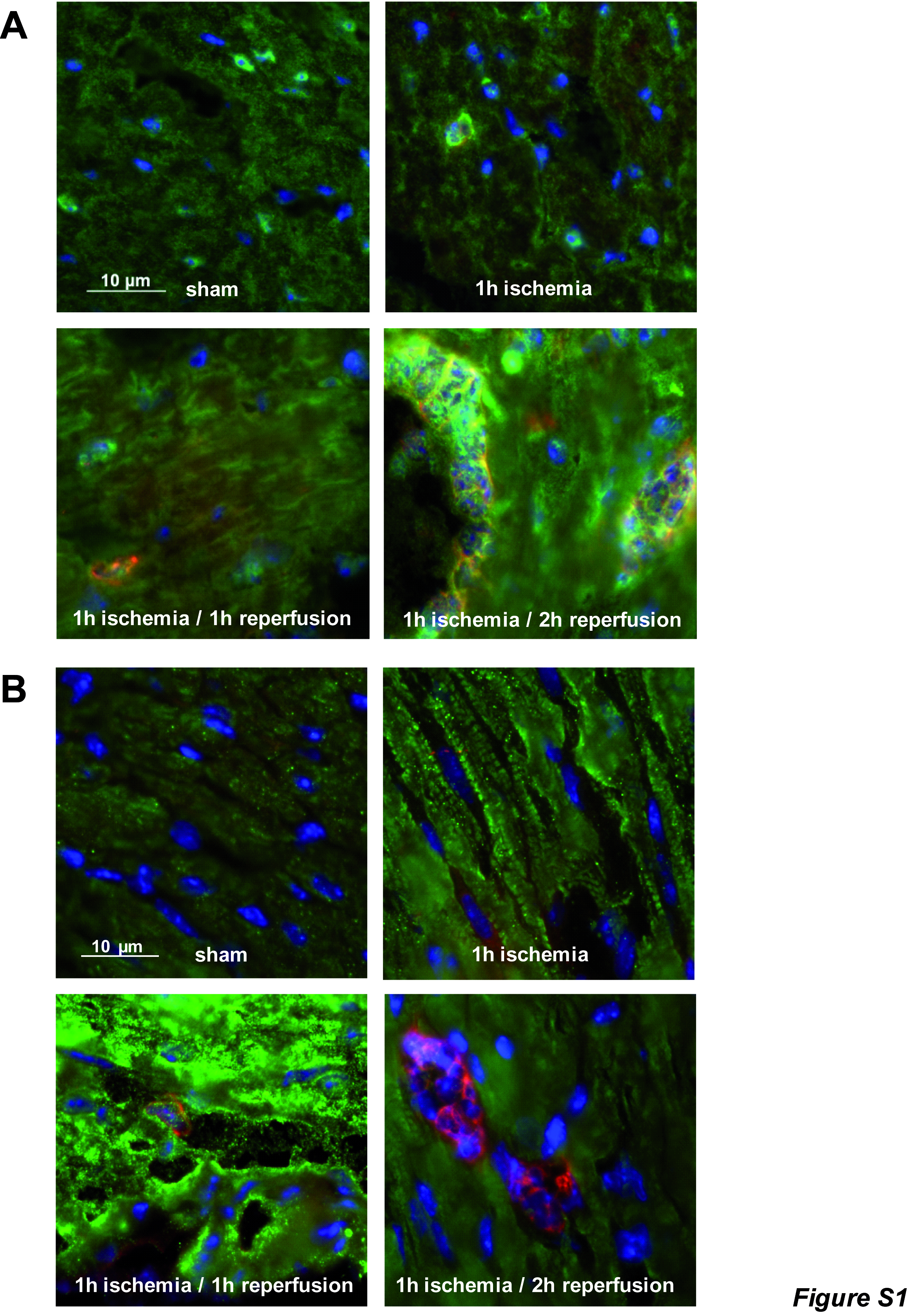

Supplement: Figure S1 — PMN infiltration in heart tissue during IR-injury. Surgeries in WT mice were performed as indicated. To stain infiltrated neutrophils, a source for the level changes in Gαi protein expression, immunohistochemistry with an anti-CD15 antibody was performed. Additionally, tissue was stained with a. Gαi2- and b. Gαi3-specific antibodies. Representative images are shown. Scale bar = 10 µm. (TIF) [file pone.0098325.s001.tif]

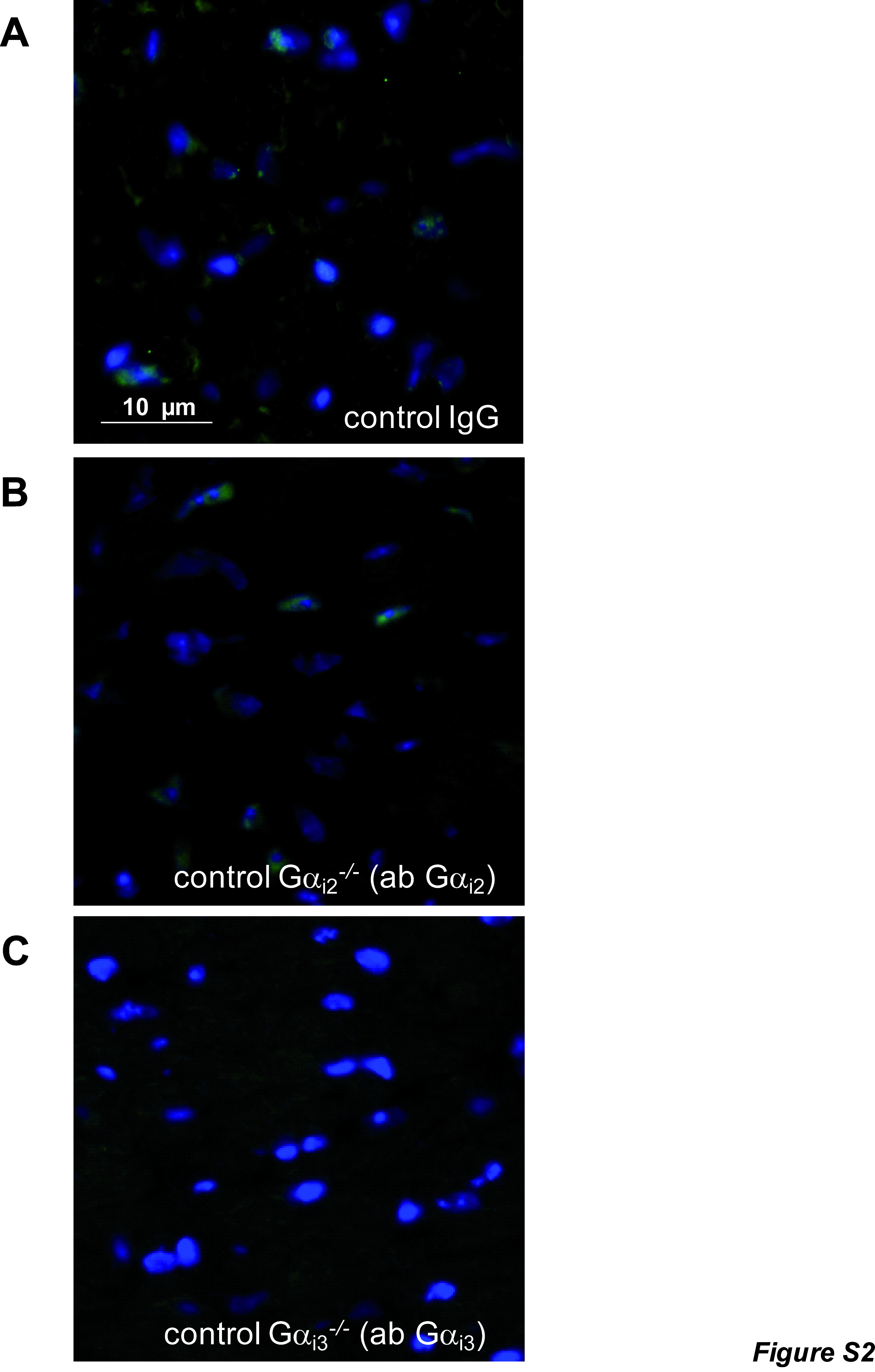

Supplement: Figure S2 — Control staining to test antibody specificity. To rule out unspecific binding of the used antibodies in heart tissue control staining were performed as follow. a. Staining of WT tissue with IgG antibody. b. Heart tissue from Gαi2 -/- mice was stained with anti-Gαi2 antibody. c. Heart tissue from Gαi3 -/- mice was stained with anti-Gαi3 antibody. Representative images are shown. Scale bar = 10 µm. (TIF) [file pone.0098325.s002.tif]

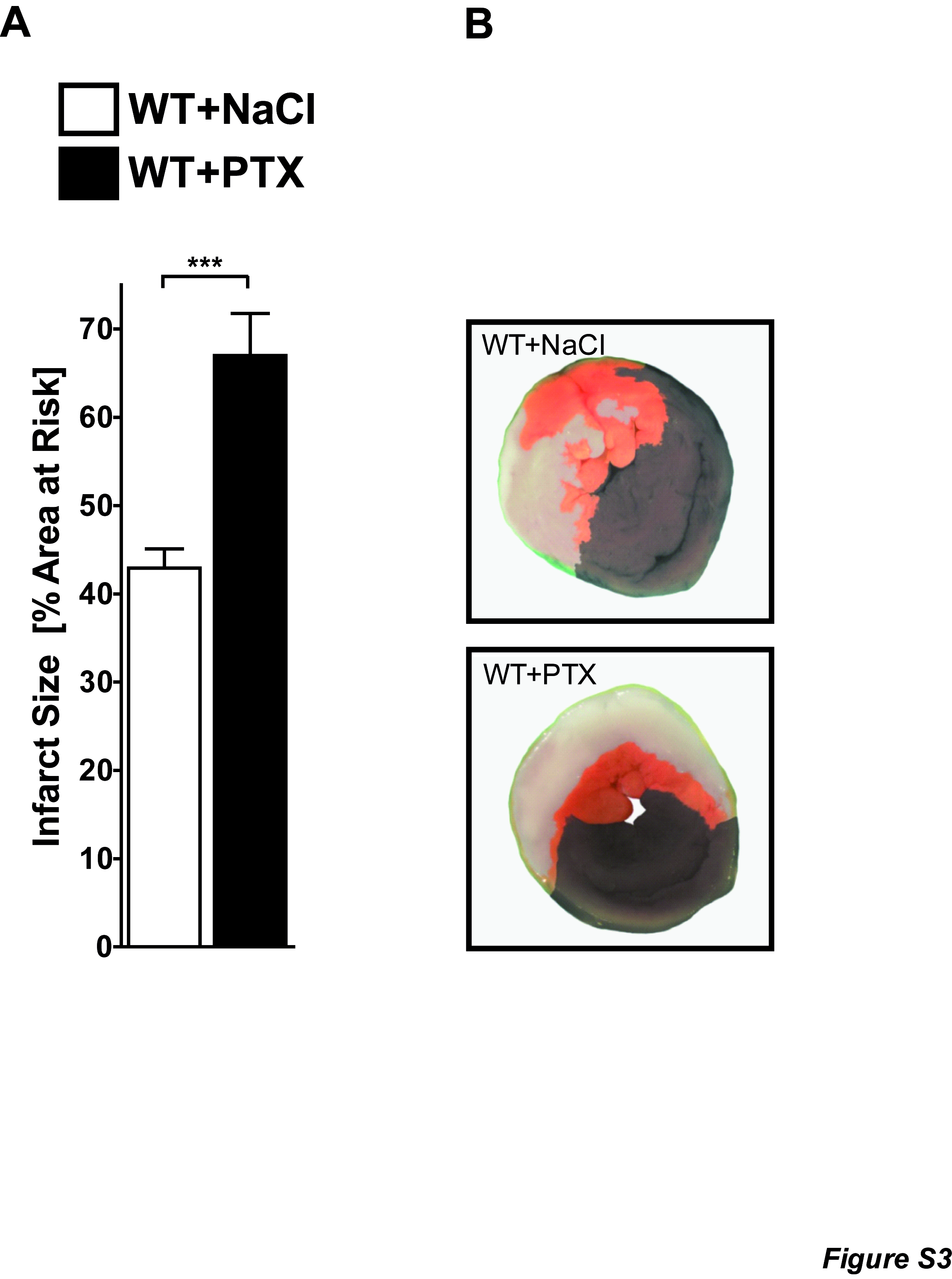

Supplement: Figure S3 — PTX treatment aggravates IR injury. a. WT mice were either injected i.p. with vehicle (n = 6) or Pertussis toxin (PTX)(see Methods S1) (n = 6) and 48 hours later exposed to one hour ischemia and one hour reperfusion. Hearts were counterstained with Evans Blue to determine the AAR and TTC to mark vital tissue (red) and necrotic tissue (white). Subsequently, infarct size was calculated as percentage of AAR. b. Representative heart slice of WT mice treated with NaCl or PTX are shown. These heart discs have an infarcted area of 46% (WT+NaCl) and 69% (WT+PTX). Data in (a) are shown as mean ± SEM; statistic was calculated with t-test; ***P≤0.001 as indicated. (TIF) [file pone.0098325.s003.tif]
