# Supplementary material for: Gαi2- and Gαi3-Deficient Mice Display Opposite Severity of Myocardial Ischemia Reperfusion Injury
Source: PLoS One. 2014 May 23;9(5):e98325. doi: 10.1371/journal.pone.0098325 (PMC4032280; doi:10.1371/journal.pone.0098325)
Supplement: Methods S1 — Pertussis Toxin treatment. (DOCX) [file pone.0098325.s004.docx]

**Supplemental Method**

**Pertussis Toxin treatment**

Mice were injected intraperitoneally with 150 µg/kg body weight Pertussis Toxin (PTX; Merck Millipore) or NaCl solution (0.9 %) as vehicle control 48 hours before surgery.

**Supplemental Figures**

**Suppl. Fig. 1: PMN infiltration in heart tissue during IR-injury.**

Surgeries in WT mice were performed as indicated. To stain infiltrated neutrophils, a source for the level changes in Gα_i_ protein expression, immunohistochemistry with an anti-CD15 antibody was performed. Additionally, tissue was stained with **a**. Gα_i2_- and **b.** Gα_i3_-specific antibodies. Representative images are shown. Scale bar = 10µm.

**Suppl. Fig. 2: Control staining to test antibody specificity.**

To rule out unspecific binding of the used antibodies in heart tissue control staining were performed as follow. **a.** Staining of WT tissue with IgG antibody. **b.** Heart tissue from Gα_i2_^-/-^ mice was stained with anti-Gα_i2_ antibody. **c.** Heart tissue from Gα_i3_^-/-^ mice was stained with anti-Gα_i3_ antibody. Representative images are shown. Scale bar = 10µm.

**Suppl. Fig. 3: PTX treatment aggravates IR injury.**

**a.** WT mice were either injected i.p. with vehicle (n=6) or Pertussis toxin (PTX)(see supplemental method) (n=6) and 48 hours later exposed to one hour ischemia and one hour reperfusion. Hearts were counterstained with Evans Blue to determine the AAR and TTC to mark vital tissue (red) and necrotic tissue (white). Subsequently, infarct size was calculated as percentage of AAR. **b.** Representative heart slice of WT mice treated with NaCl or PTX are shown. These heart discs have an infarcted area of 46 % (WT+NaCl) and 69 % (WT+PTX). Data in (a) are shown as mean ± SEM; statistic was calculated with t-test; ****P* ≤ 0.001 as indicated.
